# Causal allocation of fixed impacts in product systems: Assessing the effect of data demand on network energy consumption

**DOI:** 10.1111/jiec.70057

**Published:** 2025-07-12

**Authors:** Daniel Schien, Paul Shabajee, Louise Krug, Greg McSorley, Chris Preist

**Affiliations:** 1 School of Computer Science, University of Bristol, Merchant Venturer's Building, Woodland Road, BS8 1UB Bristol, UK; 2 BT R&D, Ipswich, UK

**Keywords:** causal modeling, consequential LCA, environmental assessment, environmental sustainability, Internet energy consumption, telecommunication networks

## Abstract

Environmental assessments of digital services currently apply an accounting perspective, and for telecommunication networks (TN) allocate electrical energy consumption in proportion to data traffic. Yet, the power draw by wired TN infrastructure is almost independent of the volume of data traffic flowing through it. Previous assessments of the effect of data traffic on energy consumption thus tended to over-estimate the short-term impact on energy consumption.

However, the growth of *peak* data traffic rates is a main driver of increasing TN bandwidth capacity and has an indirect impact on electrical energy consumption. This nuanced causal relationship has not been consistently represented in allocation approaches used for attributional carbon footprints.

In this text, we apply a form of consequential system expansion by considering the long-term response to peak-traffic growth. This allows us to model long-run marginal changes to product system attributes that are fixed in the short-term. The outcome illustrates a causally consistent allocation approach that avoids contradicting the short-term behavior of the engineered system.

Based on a causal inference graph of the drivers for the fixed baseload power draw by TN, we distinguish between the effects of different types of data as they contribute to traffic peaks. From this, we develop transform functions that re-allocate environmental burden to peak traffic. We present such functions for the specific case of periodically diurnal traffic in TN (including video-on-demand) and discuss the case of sporadic high-throughput events (including video streaming of life sport events and games downloads).

The allocation model incentivizes a reduction of peak demand through avoidance or demand-shifting, to decelerate the long-term expansion of TN infrastructure.

## INTRODUCTION

Large-scale digital services such as video conferencing, streamed entertainment, social media, remote office working, and generative AI are widely used globally and have significant environmental impacts (Carbon Trust, 2021; Luccioni et al., 2024; Widdicks et al., 2022). Robust environmental assessments of these services can support policy making, focus innovation, and guide sustainability strategies (including setting of targets) by the content service provider (CSP) companies that supply them.

Such large-scale digital services are delivered via interoperating networks that span the globe, owned and managed not by the CSPs but by telecommunication network operators (TNO) and Internet service providers (ISP). Part of the environmental impact of each digital service is therefore a share of the impact of the network infrastructure and the electrical energy (hereafter referred to as *energy*) used to operate it. Hence, a key methodological question in the assessment of digital services is how best to allocate a share of network energy use to each service using it.

When evaluating the environmental impact of product systems with life cycle assessments (LCA), some processes vary directly in proportion to demand, while other impacts are fixed and change only over the long term. The balance between variable and fixed impacts varies between product systems. However, both are important to address as part of carbon emission mitigation.

This is particularly important when LCA is playing a role in corporate policy and aspects of decision making, for example, target setting and procurement via product carbon footprints (PCFs) (GHG Protocol, 2011; Karlsson & Luttropp, 2006).

Currently, the most widely used model for allocating energy consumption (and consequent “use phase” carbon footprints) from network use to services provided over a network, attributes a share of total annual network energy consumption in proportion to the share of the total data volume transferred.

However, neither the power nor energy consumption of network devices is directly proportional to the data volume transferred over them; instead, their power consumption is characterized by a high “always-on” baseload (or “static”) power draw that is determined by the maximum *capacity* (called “bandwidth”) and an additional variable component that changes with the *utilization* of the device for data transfer, the so-called “throughput.” The ratio between baseload and dynamic portion of power consumption is different between wired^1^ (optical and electrical) and cellular (radio) network devices. For the former, which we focus on here, the baseload power draw represents 80% or more of the maximum power consumption (Chan et al., 2016).

This means that changes in the transferred data volume do not affect energy consumption in the infrastructure transferring that data; it is not “energy proportional” relative to the highly variable data transfer demand (Barroso & Hölzle, 2007). The prevailing use of energy intensity coefficients for allocation in environmental assessments, therefore, is predicated on a causal relationship that does not exist. Above all, such metrics cannot be used to estimate changes in energy consumption from changes in data volume being transferred (Schien et al., 2024).

In this paper, we argue that, despite this lack of instantaneous causality, there *are* relevant causal relationships between network usage and energy demand, which can support the design of allocation metrics. However, these occur over longer time periods of months and years, rather than instantaneously. To understand these, we need to look at the drivers of network expansion and how usage of the network influences these. In particular, we will consider how usage at peak times can drive the expansion of the network capacity.

In Section 2, we review the existing approach to the allocation of network energy use, identify the problems allocation faces due to the non-energy-proportionality of network devices, and the resulting methodologically inappropriate application in the literature. In Section 3, we use causal influence modeling to develop an understanding of the longer-term drivers of network expansion and hence energy use in the network. In Section 4, we develop a family of allocation functions based on this understanding, which can be used in LCA to more effectively apportion the burden of network impacts in a way that reflects the underlying longer-term causality of energy use increase. Hence, such functions will apportion responsibility to different services more effectively than the current approach.

Finally, in Section 5, we discuss the implications and practicalities of our approach and also consider how the approach could be applied to other aspects of digital infrastructure.

## BACKGROUND

### Carbon footprinting of digital services

Methodologies from environmental impact assessment have been applied in an effort to understand, quantify, and reduce environmental impacts of digital technology and the services it provides. Environmental LCA has been used to assess the impacts of digital hardware, such as (Busa et al., 2019). A restricted form of this, focused only on global warming potential, is also used to assess the “carbon footprint” (Pandey et al., 2011).

**FIGURE 1:**
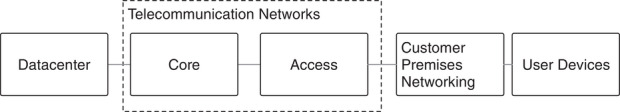
Parts of the minimal product system for distribution of digital services. In the focus of this text is the core layer of telecommunication networks.

Much of today's digital economy is equally focused on the provision of digital services, and such assessment techniques have also been applied to these. Examples of PCFs for digital services include Achachlouei et al. (2015), Schien et al. (2021), Weber et al. (2010), and Williams and Tang (2013). These consider how individual digital services use different parts of the IT infrastructure, as illustrated (in simplified form) in Figure 1.

The Greenhouse Gas Protocol (Carbon Trust & Global e-Sustainability Initiative (GeSI), 2012) uses carbon footprinting techniques to retrospectively report on emissions associated with companies and the products/services they provide. In particular, companies are encouraged to report emissions beyond their organizational boundaries that are associated with the creation and delivery of their products and services (referred to as scope 3 emissions). For companies providing digital services over the Internet, emissions associated with IT infrastructure used but not owned by the service provider can fall within the corporate reporting boundary of scope 3 emissions. For example, streaming media companies often report estimates of their services' use of core and access networking infrastructure owned by various ISPs around the world (DIMPACT, 2024).

In this text, our particular focus is on how to appropriately allocate emissions associated with fixed networks owned by the ISP to the digital services that use them (for PCFs) and the companies that provide those services (for scope 3 or PCF emissions reporting). In the discussion section, we will expand our consideration to other elements of the IT infrastructure.

Environmental assessments serve a variety of goals (Weidema, 1998). These can be “informational,” such as marketing claims, consumer information, and voluntary or statutory reporting. These tend to be retrospective, reporting estimates of past emissions.

Alternatively, they can be “change-oriented” such as policy development, product design choices, procurement choices, behavior change advice, and hot spot elimination. These tend to be prospective assessments, forecasting future emission changes under different scenarios.

LCA methods offer two complementary approaches—attributional, and consequential (Sonnemann & Vigon, 2011). The attributional approach aims to quantify the environmental burdens of the processes that contribute to a given system under study and allocate them all to the products and services it produces. It can be used to identify hotspots where action would be most beneficial and assign responsibility for emissions to different parties (Brander, 2019). The consequential approach aims to quantify the change in burdens resulting from a change in the system. Both approaches have a role in decision-making. While there is debate regarding the relative roles of the two approaches (Finnveden et al., 2022; Weidema et al., 2019), there is broad agreement that the “widespread misuse of attributional methods for decisions about actions” (Brander, 2019) and to quantify their effects is problematic.

With regard to digital services, there is significant use of attributional analyses in this way. Almost universally, they use a metric for allocating energy use (and therefore use phase emissions) from networks to the various services that run across them according to the quantity of data that are transferred. As a result, they advocate reducing emissions by reducing the quantity of data transferred by the service, so that their contribution to network infrastructure usage is reduced. For example:

- Website designers are encouraged to reduce the size of web pages. Advocates claim this will reduce the carbon footprint of their use in direct proportion to that reduction (e.g., 59 Wh/GB proportional change of energy consumption in the network (Wholegrain Digital, 2025))
- Video conference users are encouraged to turn off the video and use audio only. Advocates claim this will result in significant emissions saved. For example, Obringer et al. (2021) claim that if 1 million videoconference users were to make this change (on 15 1-h video calls per month), they would collectively reduce emissions by 9023 t of CO^2^e in 1 month.

While these approaches are well motivated, the claims of emission reductions they make are wrong and based both on incorrect methodological reasoning and on a misunderstanding of the technical dynamics of the system. Furthermore, even as an attributional claim of responsibility, they are inadequate, as we will explore further in the following sections.

Furthermore, initiatives such as the Science Based Targets Initiative (SBTI, 2023) encourage companies to commit to reduce their (retrospective) reported Scope 3 emissions over time. For digital services using internet telecommunication networks, under the currently widely used approach used to allocate emissions, one way of reducing reported Scope 3 emissions is to reduce the amount of data a service uses over the internet. A consultancy industry is building around advising companies how best to do this (e.g., (Engie Impact, 2024)).

Hence, individual, corporate, and governmental behavior with regard to digital service emissions reductions is influenced by the metric chosen to allocate emissions from networks to services, and claims of emissions reductions are made based on the existing allocation metric. In the following subsection, we will demonstrate that it is not fit for purpose for use in this way.

### Allocation of network energy consumption

When conducting environmental assessments of industrial systems, it is recognized that processes that supply more than one product are challenging, and the decision of how to allocate the environmental burdens associated with these requires careful consideration (Sonnemann & Vigon, 2011). In the context of this paper, our concern is the apportioning of environmental burdens associated with the provision of networks, to the digital services (such as video and audio streaming services, social networks, and websites) that run across it (and for scope 3 reporting, to the companies that provide those services.)

For fixed telecommunication networks, the most common allocation approach has been to share energy among traffic flows in proportion to data volume transferred via an energy intensity metric $I_v$ (Hossfeld et al., 2024) (e.g., measured in kWh/GB). With this metric, the energy $E$ can be described as a *linear* function of the data volume $v$ of the service for which the carbon footprint is being calculated (Equation 1): $E = v \cdot I_v$. For access networks, time has sometimes been used to estimate energy consumption, as in $E=t \cdot I_t$ (Schien et al., 2013). Then, $I_t$ is equivalent to allocating a share of the power consumption of the infrastructure to the duration of use $t$ (e.g., x kWh of energy per hour of use). This could be the time for watching a movie.

The estimation of these coefficients has as long a history as their use in estimating energy consumption of digital services, with Koomey et al. (2004) and Taylor & Koomey (2008) being among the earliest. These intensities have been estimated via top-down or bottom-up approaches (Schien & Preist, 2014a, 2014b). A bottom-up model sums the energy intensity of network devices along an average route through a network to estimate the energy intensity of network use (e.g., Baliga et al., 2009). The majority of these assessments are desk-based using industry-average data. A notable exception is Coroama et al. (2013), who carried out a case study of a 40-megabit-per-second videoconferencing transmission between Switzerland and Japan. A top-down approach estimates the energy consumption of an entire system over a time frame (normally a year) and divides this by the total data volume transported during this time (e.g., Schien & Preist, 2014a).

Aslan et al. (2018) provided a review of intensity values and an estimated global average trend over time. Such trend analysis is valuable, as it quantifies aspects of how efficiency is changing over time. Both the GHG Protocol ICT sector guidance for assessing carbon footprints of digital services (Carbon Trust & Global e-Sustainability Initiative (GeSI), 2012) and ITU (2012) recommend using these kinds of energy intensity values as the default method for allocating energy use in fixed networks^2^ to the digital services that use them.

This approach is very widely used and superficially appears similar to other uses of allocation, where the burden of a process is shared between the users of that process in proportion to how much each uses it. If a sheet metal plant produces both car and airplane components, it makes sense to share the burden based on the proportion the plant is used to make each. This is in line with LCA standard guidance that, where allocation is necessary, it should preferentially be partitioned based on the “determining physical causal relationship” (Sonnemann & Vigon, 2011) and that allocation “should reflect the way in which the inputs and outputs are changed by the quantitative changes the[…]functions are delivered by the system” (International Organization For Standardization, 2006). There is an implicit assumption that the use of networks follows the same *physical causal relationship* between how much data are transferred by a given device and how much energy is used to do this. This is not the case. Networks have a low energy proportionality (Barroso & Hölzle, 2007): The power draw of network devices is largely independent of throughput at any given time but rather is determined by their maximum bandwidth capacity (Cisco, 2022).

For this reason, the application of intensity metrics (kWh/GB) to estimate avoided or increased carbon emissions in proportion to data traffic has been widely critiqued, particularly when used to make change-based claims (Carbon Trust, 2021; Koomey & Masanet, 2021; Malmodin, 2020; Mytton et al., 2024). The linear energy intensity coefficient (e.g., kWh/GB) implies that the power consumption $P_T$ of a system is a linear function of the data volume that it transports. However, because network devices have a low energy proportionality, the power consumption is better understood as a function that is the sum of a static baseload power consumption $P_b$ (in Watts), which is independent of use, and a dynamic portion that scales with utilization $P_u$ (also in Watts), such that $P_T = P_b + u\cdot P_u$. For network devices, baseload power is 80%, or more, of total power consumption (Chan et al., 2016).

**FIGURE 2:**
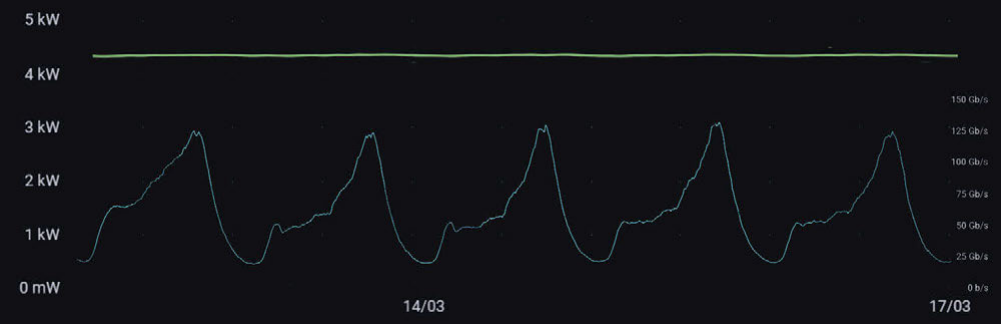
Illustrative measurement of power draw against throughput of a carrier-grade service router deployed by BT over 5 days. The nominal energy intensity varies in response to demand between approximately 0.55 and 0.075 kWh/GB. The underlying data for this figure are available via the repository mentioned in the data availability statement.

This lack of correlation between throughput and instantaneous power draw is illustrated in Figure 2 for a typical type of core network device (here a network switch), which shows very little variation in power draw despite large changes in utilization, for example, intensity variations between approx. 0.074 and 0.58 Wh/GB over the day (as illustrated below as simple direct allocation); note that these are operational intensities rather than optimal intensities that the device is capable of.

Hence, widely used linear intensity coefficients $I_v$ falsely imply that energy consumption is proportional to data throughput. This means that allocation using such a coefficient does not use a “determining physical causal relationship,” the preferred approach to allocation in LCA standards.^3^ As a result, the instantaneous and longer-term dynamics of power consumption and utilization are currently not adequately treated in environmental assessments (Schien et al., 2024). Specifically, the quantified claims regarding reductions in network energy use from shrinking web pages and turning off video cameras described in 2.1 are not correct. The data presented above show that, in the core network, it will not result in a reduction in energy use in the deployed device.

Because of these problems, an alternative approach has been proposed by Malmodin (2020) and Mytton et al. (2024). They propose a model that introduces a constant, per-subscriber portion of fixed baseload power draw $P_b$ (in Watts) that is combined with service use time and calculate an intensity metric $I_{v}^{d}$ (e.g., in kWh/GB) to allocate only the dynamic power consumption in proportion to data volume in Equation (2): $E = P_b \cdot t + v \cdot I_{v}^{d}$. This approach offers a better representation of the (small) *instantaneous* change in energy consumption from a change in transported data volume. However, that model does not improve on the more substantial problem of allocating baseload power consumption to core network use in a way that reflects a determining causal relationship. By making baseload independent from data transfer, the model simply uses an alternative proxy factor, subscribers (or a number of end-user devices), which are similarly causally decoupled from the underlying baseload energy use in wired core networks. La Rocca et al. (2024) study the aggregate energy consumption of a transmission network. Similar to us, they consider peak throughput as the main determinant of energy consumption for network devices. They estimate network energy consumption over a range of scenarios of peak traffic rates, such as HD versus UHD streaming, and contrast this to the energy consumption in a network designed to provide only “minimalistic” levels of service. As their model applies aggregate peak traffic as a composition from all services, it requires an additional allocation, if it is to be used to calculate a service provider's scope 3 emissions for individual digital services. In its current form, it only estimates aggregate energy consumption necessitated to provide bandwidth for the whole set of services that jointly constitute peak traffic.

To develop methods to allocate the baseload more appropriately to individual service use, we now consider what causal relationships exist between network “usage,” and network energy consumption. What drives changes to energy consumption over time?

## A SYSTEM MODEL OF DATA TRAFFIC AS A DRIVER OF NETWORK CAPACITY AND BASELOAD POWER

Currently and in the past, at any point in time, baseload energy use of network infrastructure is a function of bandwidth capacity (as well as other factors). That is, for any product range of device, those with higher capacity will tend to have higher baseload power consumption (Cisco, 2022). In addition, over time, new devices are becoming more energy-efficient in transferring data as capacities increase. However, in practice, old lower-capacity devices will be replaced with new devices with higher capacity and improved energy efficiency at peak load. While the new device will be more energy efficient at its peak operating capacity, its baseload power consumption may be relatively similar to the older device. This means it may not be significantly more efficient at the level of utilization when it is installed. The efficiency gains may only show as the demand for capacity increases.

**FIGURE 3:**
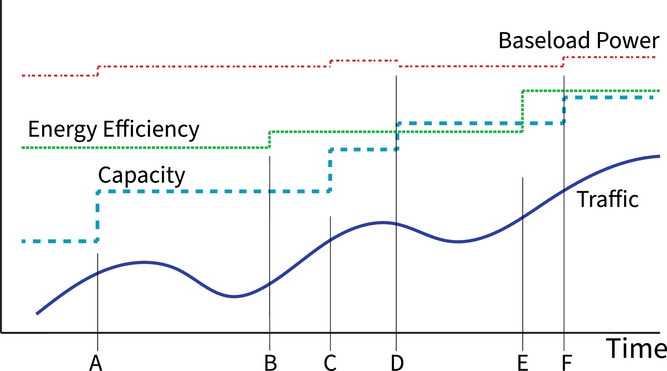
Scale-free, illustrative chart for a single network device of dependent change over time of traffic (solid, blue), baseload power draw (top line red, dashed), energy efficiency of devices (green, dash middle), and capacity (blue, lowest dashed line) of a fixed Internet service provider (ISP) network. As service demand drives peak traffic, additional bandwidth (capacity) is provided by replacing network devices when throughput nears capacity thresholds (at time A, C, D, and F). Baseload power changes when devices are replaced (also at times A, C, D, and F). Energy efficiency of devices improves (increases) over time and independently of the deployed network, as devices come to market (at times B and E). Baseload power is a function of capacity and energy efficiency and can go up (at times A and D), or down (at times C and F). Not shown here are impact from manufacturing of devices (i.e., embodied impact) and the dynamic power consumption, which contributes to a relatively much smaller degree to overall power consumption.

The combination of dynamics as illustrated in Figure 3 shows a simplified view of how peak demand drives capacity and in turn power consumption in a single network device. As the peak traffic from services reaches the operational bandwidth capacity of network devices, additional capacity is provided by replacing or enhancing them. After upgrades, the baseload power consumption steps up, or possibly down, if the efficiency improvement of the new device is relatively greater than the increase of the newly provisioned capacity. The dynamic power consumption contributes to a relatively much smaller degree to overall power consumption.

At the level of the whole network (including core and access networks), the dynamics are even more complex and hidden when aggregate metrics are used.

**FIGURE 4:**
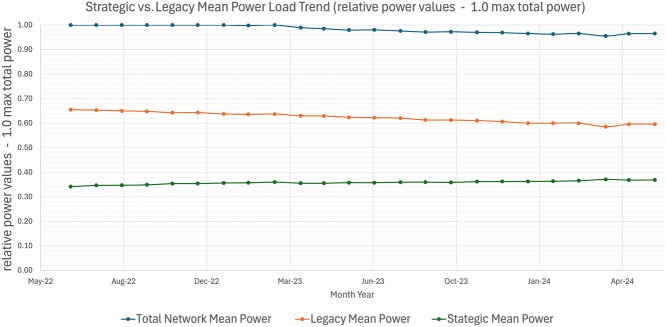
Trend in energy mean power use over time (June 2022–May 2024) of BT Strategic and Legacy Networks. The underlying data for this figure are available via the repository mentioned in the data availability statement.

National-scale telecoms networks are complex and composed of both current “strategic” networks that carry the bulk of traffic and legacy network technologies that are being phased out, for example, in the United Kingdom, the 40-year-old Public Switched Telephone Network telephony network. Figure 4 illustrates this for the case of the BT Network (including OpenReach), providing much of the United Kingdom's core and access network infrastructure.

While the overall energy use is seen to be declining between March 2022 and January 2024, this trend is composed of both kinds of networks. Energy consumption in the legacy networks is shrinking as sites are being closed and devices removed as the services are no longer being supported. While the strategic energy use has varied over the period, the overall strategic platform trend has seen slow growth (approx 3.5%) despite several cycles of local device replacement. Drivers such as efficiency improvements of devices (e.g., from optimization and miniaturization) help to reduce power draw but have not been sufficient to offset the growth of traffic. The long-term trend is growth of about 2%–4% over about 15 years. It is currently at the high end of the range, because of fiber to the premises (FTTP) deployment (major access network uplift) and is projected to slow to around 2% pa by 2030 (BT R&D, 2024)

Importantly, legacy removal is not a continuous process as legacy infrastructure can only be removed once. Given the current IP-based networking paradigm (short for “Internet Protcol”), when the legacy systems have been removed, the remaining strategic network will not become “legacy”—even though devices within it will carry on being upgraded. Even as full fiber deployment is completed, projections based on current trends in peak bandwidth growth yield the expectation that the growth of energy consumption will continue, albeit at a lower rate.

We argue that networks could have a lower power consumption (from baseload) or rate of growth, if the peak traffic was lower. Energy consumption of network devices is defined by their peak throughput. Notwithstanding background efficiency improvements and assuming similar functionality, a network device with lower peak capacity will tend to have a lower power consumption (e.g., Cisco, 2022). It is thus the required capacity to satisfy traffic peaks that determines the device class and informs its base and dynamic energy consumption. (Additionally, but not primarily in this investigation, upgrading devices drives more cumulative GHG emissions through the impact from manufacturing, transport, and installation of the new devices.)

The current allocation approaches described in Section 2.2, therefore, omit these important dynamics of the underlying system. They are not based on a “determining physical causal relationship” (Sonnemann & Vigon, 2011, p. 277) and are not “reflecting effects of change” (Tillman, 2000). In some ways, this is understandable. Both are straightforward to develop with aggregate data and assume an immediate causality that often exists in physical product systems. However, power draw by network devices is fixed in the short term, and the causal relationship between data throughput and energy consumption manifests over the longer term, over months and years. We now consider how such longer-term causal relationships can be reflected in an approach to allocation through an explicit consideration of factors within the wider dynamic system in which they reside (Jackson, 2000; Sterman, 2014).

**FIGURE 5:**
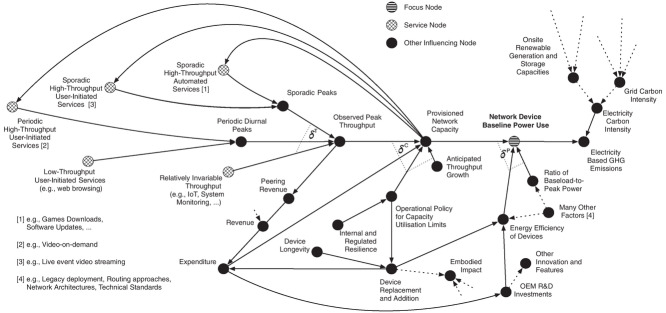
Simplified causal influence graph of fixed baseload power consumption by telecommunication network devices, focusing on the influence of types of traffic. All factors are influenced by multiple constituent factors with varying degrees ($\delta _i$) of strength. This is illustrated by “overall peak bandwidth demand” being driven jointly by “sporadic peaks” and “periodic diurnal peaks” with individual degrees $\delta _i^T$.

In Figure 5, we present a causal influence graph developed through a series of expert consultations (BT R&D, 2024). This makes explicit, and enables *scrutiny* of, assumed cause–effect flows in the system. It models the key factors and causal relationships influencing “network device baseload power use.”

The fixed “network device baseload power use” is driven by the “provisioned network capacity” as a function of peak demand and the “ratio of baseload-to-peak power,” and the “energy efficiency of devices.” Through the diagram, we can trace the influencing factors and their constituents. For example, “overall peak throughput” is composed of three categories of data use: traffic with a periodic diurnal pattern (e.g., video content, web browsing), relatively invariable traffic (e.g., IoT), and sporadic event-based traffic (e.g., live sports video streaming). The capacity also depends on network operator policies on “capacity utilization limits” (utilization levels beyond which devices are upgraded), which are constrained by “resilience” requirements and drive “device replacement and addition.” The replacement rate drives the fixed embodied impact (which is left abstract). “Revenue” from traffic supports “expenditure” into “provisioned network capacity” benefiting in turn “R&D investment” by device manufacturers, some of which contributes to increased “energy efficiency of devices” *available in the market*. “Device longevity” (e.g., low failure rates and ongoing security updates) allows keeping devices for longer, which lowers both “expenditure” as well as “energy efficiency of devices” *in the fleet operated*.

Such a model allows one to:

- identify possible *points of intervention*, their interaction, and associated stakeholders. For example, the diagram shows the causal connections between “internal and regulated resilience” and “network device baseload power use,” and hence, stricter regulations can increase energy consumption.
- through including temporal relations, make temporal dynamics explicit, for example, impacts manifesting with delays due to device lifetimes.
- identify potential (negative and positive) unintended consequences (such as rebound effects) through feedback loops and interactions with other technical and socio-technical systems. For example, the directed link between “provisioned network capacity” and “sporadic high throughput user initiated services” shows that higher available capacities might enable the development of even higher bandwidth services such as cloud video gaming.

## CAUSAL ALLOCATION APPROACHES TO FIXED BASELOAD ENERGY

We now apply the causal influence model described above to design allocation metrics that allocate fixed energy consumption within networks to the services that use them. By applying systems thinking we follow LCA methodology and ground the design of allocation approaches on principles of causality. The overarching approach applied here takes the following steps:

1. Identify and model the causal factors that drive the energy use and/or GHG emissions (and/or other metrics) and their interactions over causally appropriate timescales.
2. Study the interactions within the system and derive allocation principles based on the change of the system as constrained by limiting factors.
3. Weighting (“cost”) the force of the drivers based on their causal significance in the given context, to prioritize or derive quantitative allocation models.

The model shows that if we are to reflect the longer-term causal relationships, such an allocation metric should account for the extent usage at any given time, drives increases in observed peak throughput (which in turn drive upgrades in provisioned network capacity). Specific “peak throughput” events result from a *coinciding* of (i) diurnal peaks driven by periodic user behavior, which are steadily increasing in size; (ii) specific “event peaks” such as the release of a major videogame or a live streaming of an international sporting event, for example, the Women's Football World Cup 2023 (Jackson, 2022, 2023a, 2023b, 2023c, 2024).

We present an allocation approach focused on a quantitative analysis of *periodic diurnal traffic*, which better reflects the causal relationship between such diurnal peaks to the longer-term expansion of the network (and hence increase in energy use.)

We draw inspiration from the “use of system” pricing models related to infrastructural overheads in energy grids (Australian Energy Regulator, 2021). Such models are also policy instruments that incentivize demand-shifting behaviors to reduce energy consumption at peak times with the explicit goal of constraining growth and reducing the need for infrastructure development and thus costs (including carbon emissions). For example, in the UK grid transmission system, the DUoS and, historically, TNUoS charge systems consider both diurnal and annual peak utilization intervals in a given year and distribute high charges to consumers during these periods (National Grid ESO, 2022). Time-dependent pricing for consumers has also been applied to networks, in particular mobile networks (Chiang, 2012).

Similarly, our approach burdens traffic at peak time with a higher share of the baseload energy consumption than traffic at other times. We present a family of illustrative functions that weigh the impacts to different degrees and begin to explore which is more appropriate. In general, such functions require statistical or operational data for a specific network. For this study, we illustrate the principles on observed data from early March 2024 of a single deployed router in the UK core network.

**FIGURE 6:**
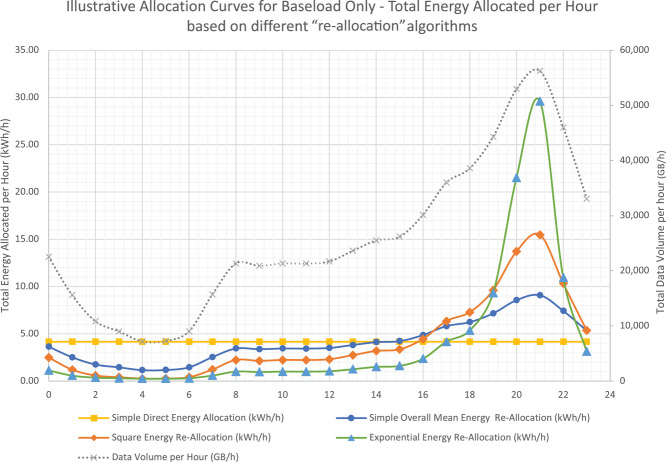
Total energy per hour allocation based on the different re-allocation algorithms and data volume (demand) per hour. The underlying data for this figure are available via the repository mentioned in the data availability statement.

We begin by exploring ways of “re-allocating” energy consumption toward the peak utilization periods, that is, toward our target causal driver identified from causal modeling, before deriving allocation models. We resolve time in hourly periods, although monitoring of both energy use and data traffic could be at higher resolutions. Figure 6 shows traffic (dotted gray line in units of data volume GB per hour) and baseload energy use (shown by the constant yellow line in kWh per hour).

To this, we apply transformation functions to the data traffic per hourly time window $V_i$ (e.g., in GB) that allocate a weighted share (here $\delta _2^T$ for “periodic diurnal peaks”) of energy (kWh) across the day and normalize to ensure the total energy allocated remains constant per Equation (3).

3 $$ E^r_i = \delta _2^T \cdot E_i \cdot \frac{f(V_i))}{\sum _{i=1}^{24}f(V_i)} $$

We apply the following illustrative allocation approaches, each increasing the degree of reallocation toward the peak. Other functions, such as an S-curve are possible.

1. Direct energy allocation without transform: $E_i = E_i$ (equivalent to hourly baseload energy consumption)
2. Direct proportional re-allocation - $f(V_i) = V_i$
3. Squared re-allocation - $f(V_i) = V_i^2$
4. Exponential re-allocation - $f(V_i) = e^\frac{V_i}{\mathrm{min}(V_i)}$

**FIGURE 7:**
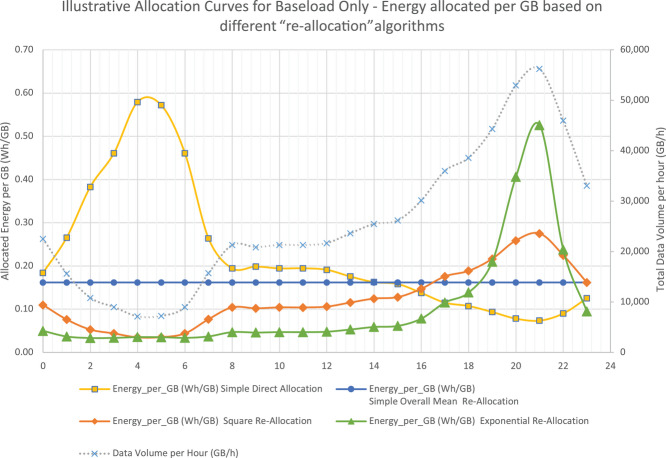
Energy per GB in each hour, allocation based on the different re-allocation algorithms, and data volume (demand) per hour. The underlying data for this figure are available via the repository mentioned in the data availability statement.

We then derive allocation models by dividing the reallocated energy consumption by the data volume in the hourly interval $I_{i}^{^{\prime }} = {E^r_i} / V_i$ in units of energy per data volume (kWh/GB in this illustration). These intensities are shown in Figure 7.

As can be seen, the transformations reallocate energy per unit of data volume (per GB) around the day depending on the utilization of the network at that time to differing degrees. Strikingly, the simplest function (1) “simple direct energy allocation,” which might naively (i.e., with limited causal understanding) be thought of as more “real-time,” and so more “accurate,” very strongly shifts the burden per GB transferred from peak times to those at low utilization periods. This is the inverse of a demand-causal metric. This model would incentivize shifting traffic *toward* peak utilization rather than periods of lower demand. Interestingly, the current practice of allocating a uniform energy per GB to all times of the day mirrors transform 2 “direct proportional reallocation.”

As might be expected, the “steeper” squared and exponential reallocation functions further amplify the burden toward data transferred during the peak period and more strongly away from the periods of low utilization. A more extreme version could be a step-function, allocating all energy only to the peak hours.

A causally based choice of a specific transform function would be to develop and choose the function based on the degree and ideally detailed dynamics of causal influence on our focus factors, that is, baseload energy use and overall carbon emissions.

We have illustrated the causal approach for the case of periodic daily peaks. This approach can also be used to analyze longer time scales and the significant but sporadic event peaks that occur within them. Given that historically any given year includes at least one record peak event, a window of a year may offer a suitable longer-term perspective.

## DISCUSSION

The work we have presented above gives a means to allocate energy usage in the core of fixed networks in a way that follows the underlying causal drivers of increased energy usage. This is in line with LCA recommendations that allocation be done in a way that is based on “determining physical causal relationships.” We have done this by developing an illustrative causal model of the underlying drivers of baseload power consumption, using this to identify the importance of peak usage on driving energy usage over time and developing a family of allocation functions that provide increasing levels shifting the environmental burden of usage toward these peak periods.

We outline how this approach can be used for both diurnal peaks by taking a daily time period, and longer-term sporadic event peaks by taking a yearly one. Further work is needed to identify the appropriate weighting (delta in Equation 3) between these two, and whether other factors are significant enough to warrant inclusion. For example, substitution effects between video-on-demand and live events may reduce the size of peak events. Our causal allocation approach is also applicable to assigning embodied impacts from resource extraction, manufacturing, etc., to the “cradle to gate.” Embodied impacts differ from baseload energy in that the former are sunk and amortized over time, while baseload energy consumption continues to accrue at a fixed rate during the use phase. Nonetheless, the same increases in network bandwidth capacity that affect baseload power also result in additional embodied impact. Hence, the drivers we identify are equally relevant to the allocation of embodied impacts.

We argue that our approach to allocation is more appropriate than the commonly used simple-mean kWh/GB allocation. It recognizes that the power draw by wired network devices is largely unchanging and more closely reflects the underlying causality of longer-term increases in energy usage by the network. Like the existing approaches, our approach is attributional and fully allocates the burden to all services, and so, in its current form (lacking calibration), cannot be used to estimate the actual impact of a change in the use of the system.

As a simple-mean allocation is attributional, it is in principle compatible with existing PCF and corporate reporting standards. It illustrates ways in which metrics can more effectively connect with the underlying causality of network usage and other factors; on core network energy usage, it more appropriately assigns responsibility for emissions to different services (Brander, 2019) in a way that encourages meaningful action. Hence, our allocation approach distributes the *current* burden to incentivize behavior in a way that is intended to reduce *future* impacts.

Returning to the examples given in Section 2.1, we can now see that the existing metric (a static mean kWh/GB) is a variation of our transform function 4 above. It effectively re-allocates energy use in the same way as function 4 leading to the static mean kWh/GB over the period over which the mean is taken (as illustrated in Figure 7). However, that choice was simply a coincidence: As outlined above, a static-mean intensity arose as a metric for network efficiency, not a deliberate metric for LCA allocation.

However, because it is a static-mean intensity, it cannot incentivize appropriate interventions in a way that reduces emissions over time, since the intensity metric does not weigh data transfers at peak times, nor as we discuss above can it be used to model energy or emissions savings from changes in practices or policies in the short term. This means that:

- Reducing the size of the web page will not reduce energy usage by 59 Wh in the network for every GB reduction of data transfer. The data we give above show that it will have almost no impact on immediate energy use. Only usage at peak times will potentially contribute (a comparatively small amount) to network expansion. Applying more weighted transforms such as our “squared re-allocation” or “exponential re-allocation” metrics to website use would therefore identify which network services to prioritize.
- Turning off video in conference calls will similarly have no immediate impact on energy use, despite the claims. The more weighted transforms would identify that reducing it at peak times may have some long-term value.

Our more weighted allocation transforms would also encourage other design practices of digital services, for example:

- Services that automatically reduce bandwidth at peak times, such as video conferencing systems, which (rather than switching off the video manually) reduce the resolution and frame-rate of images for all customers.
- Services that shift activity away from peaks, such as software patches and game updates, which automatically spot when the network is less used.

Although it identifies the value of these interventions by more appropriately allocating current burdens, our approach cannot estimate the emissions savings such measures will make. This would require a consequential analysis that considers a counterfactual regarding the speed of network expansion without such measures. It is interesting to note, and possible future work, that this type of transform could potentially, if calibrated with long-term system modeling, provide some metric or signal of long-term marginal (consequential) impacts of short-term resource usage.

A limitation of our approach is its practicality. To accurately apportion burdens requires a time series of use of the network over the period under consideration. One can imagine network providers publishing this information to allow service providers to report their scope 3 emissions associated with network usage more effectively. However, in practice, it is likely that the existing approach of estimating impacts with a constant kWh/GB will continue to be used for reporting purposes. It is important to recognize the inadequacy of the existing approach. Because it does not reflect the underlying causality of energy usage, it should not be used for apportioning responsibility, setting targets, or attempting to identify the value of any given intervention.

We have focused on the fixed network in this analysis. Further work is required to extend this type of analysis to the mobile network and to home/office access networks (also known as customer premises equipment [CPE]). The mobile network has a different and complex set of drivers for expansion (Williams et al., 2022), which warrant a causally focused exploration.

With regard to CPE, the energy consumption of these devices (similar to core networking devices) is almost unchanging. However, the causal dynamics differ significantly: Increases in energy consumption in CPE are presently driven by other factors such as seamless whole-home wireless coverage, backward compatibility of WiFi standards, and increased number of connected devices and not by increases in bandwidth. CPE also illustrates the potential for some savings; for example, when activity drops toward zero (e.g., overnight), the device can enter a lower power sleep stage, turning off unneeded components. An allocation method could penalize services that prevent CPE from entering such sleep states.

The potential benefits from a longer-term perspective in environmental assessment of fixed impacts apply similarly to datacenters (e.g., from baseload power consumption and embodied impacts), where growth rates of energy consumption and embodied impacts are significantly higher than in the TN sector (IEA, 2022).

When allocation models are used as policy instruments, as Merton pointed out in 1936 (Merton, 1936), such purposive action will have intended, unintended, and unanticipated consequences. In our case, the choice of allocation models as outlined above would (perhaps) drive changes such that there is a reduction in the growth of network energy and carbon impacts. However, there are likely to be various kinds of unintended impact, including on other aspects of environmental and social, business, technological, and political systems that need careful evaluation.

These latter points highlight that metrics (and the goals behind those metrics) need to be adaptive as understanding develops, contexts change, and outcomes of implementations manifest. These are difficult balances, and perhaps impossible to optimize, for example, stable business and regulatory environment required for investment versus meaningful and adaptive metrics. However, working with known limitations of causally designed, but flawed, metrics seems likely to be more effective than using metrics that we know do not map to, or account for, highly significant causal dynamics.

### Conclusions

Effective allocation of environmental impacts that do not vary in response to the provision of a product or service is challenging. In this paper, we consider wired network usage as an example. We argue that the existing allocation approach ignores the fixed impact nature of wired networks, and is often misused to justify environmental interventions that have little or no impact. We argue that an approach that reflects the underlying drivers of emissions increase over time will assign responsibility more effectively and incentivize more appropriate interventions. We present a causal influence model to analyze these drivers and identify peaks of traffic—both diurnal and in the longer term—as key drivers of network expansion and emissions increase. Using this, we develop a family of allocation functions that increases the burden on digital services using networks during peak periods. Such an approach to allocation incentivizes actions such as reducing the bandwidth of data transmission during peak periods and shifting services that are not time-critical away from peak periods. While we focus on network usage, the principles we apply could be used to investigate other fixed-impact product systems.

## ENDNOTES

^1^Also called “fixed-line” networks.

^2^The ITU notes that for mobile base stations (used in cellular networks), there is a “fixed” (baseload) and “variable” part that need allocating differently (p. 40).

^3^We note, as discussed further later, that non-causal relationships can be used in LCA allocation when a causal approach is not possible or convenient. The main issue with network energy allocation is that practitioners are treating it as causal and hence misusing it in the ways detailed in this section.

## Data Availability

The data that support the findings of this study are available in the online repository https://github.com/sust-cs-uob/JIE2023-CausalAllocation. SUPPORTING INFORMATION The numeric data for Figures 2, 4, 6, and 7 can be found at https://github.com/sust-cs-uob/JIE2023-CausalAllocation.
